# Traditional Healing, Biomedicine and the Treatment of HIV/AIDS: Contrasting South African and Native American Experiences

**DOI:** 10.3390/ijerph120404321

**Published:** 2015-04-20

**Authors:** Adrian Flint

**Affiliations:** School of Sociology, Politics and International Studies (SPAIS), University of Bristol, 11 Priory Road, Bristol BS8 1TU, UK; E-Mail: Adrian.Flint@bristol.ac.uk; Tel.: +44-117-331-0842

**Keywords:** traditional healing, biomedicine, Native American, South African, HIV/AIDS, ART

## Abstract

Traditional healing remains an important aspect of many people’s engagement with healthcare and, in this, responses to the treatment of HIV/AIDS are no different. However, given the gravity of the global HIV/AIDS pandemic, there has been much debate as to the value of traditional healing in this respect. Accordingly, this paper explores the extent to which meaningful accommodation between the biomedical and traditional sectors is possible (and/or even desirable). It does this through a consideration of Native American and South African experiences, looking at how the respective groups, in which medical pluralism is common, have addressed the issue of HIV/AIDS. The paper points to the importance of developing “culturally appropriate” forms of treatment that emphasise complementary rather than adversarial engagement between the traditional and biomedical systems and how policymakers can best facilitate this.

## 1. Introduction

This paper explores the extent to which lessons can be learned from the use of Native American and South African forms of traditional healing in the fight against HIV/AIDS, and the degree to which there is scope for meaningful collaboration in this respect in the treatment of a disease that, currently, is invariably fatal without recourse to antiretroviral therapy (ART). Based, to a significant degree, on original fieldwork conducted in South Africa, the study employs the analytic framework offered by the Native American/Indian Health Service relationship in a consideration of the South African experience. 

Native American forms of traditional healing have had, when compared with South African equivalents, a relatively long association within formal biomedical healthcare structures in the U.S. The U.S.’ Indian Health Service (IHS) acknowledged officially the value of traditional forms of healing from the mid-1970s onwards. In South Africa, this has only been the case since the mid-2000s. The U.S. experience therefore offers a useful analytic framework for an exploration of both how traditional forms of healing have been used in South Africa, and—critically—how they might be employed in future. On the basis that different healing systems can be framed and understood as being complementary to one another rather than as being in opposition to one another, the case of the IHS suggests that traditional forms of healing can offer a number of benefits to patients—even with respect to diseases like HIV/AIDS that require, on certain levels at least, some measure of biomedical engagement. 

While traditional forms of healing, both Native American and South African, are increasingly coming to be viewed as valuable by those operating within a biomedical mindset, both of these traditional systems are still largely understood to be secondary to biomedicine in their importance. Colonialism in both regions saw significant efforts by authorities to eradicate what they perceived to be practices based on superstition and irrationality and, although this binary perspective has now in some quarters been challenged, this has only become the case relatively recently. Native American healing practices were officially outlawed in the U.S. until 1973, and colonial-era laws against “witchcraft” remained in place across many African countries until the late twentieth century. It remains the case that traditional healing is generally categorised by both healthcare practitioners and policymakers as “alternative” or “supplementary”, even by those sympathetic to its basic tenets. For the most part, biomedical hegemony has resulted in traditional healing being, at best, tolerated rather than embraced by those within the establishment. Amongst many biomedical adherents, the ongoing “New Age” fascination with Native American practices in particular has served merely to re-enforce the argument that traditional healing should be viewed with a degree of scepticism. 

Policymakers are certainly now more open to the role that traditional healing can play as part of a broader approach to the practice of medicine, but the extent to which it can form an integral part of formalised healthcare—based on its own merits—continues to be much debated. While there have been calls for accommodation to be made within formal structures for traditional approaches to healing, the prescriptions as to the forms such accommodation might take have proven contentious, especially where the treatment of a disease like HIV/AIDS is concerned. Furthermore, despite (and also sometimes even because of) the efforts of countries like South Africa to offer a degree of equivalence for traditional forms of healing, it remains the case that biomedical and traditional perspectives are often presented as being competing (and often incompatible) systems. 

Accordingly, against the backdrop of HIV/AIDS, a disease that continues to threaten the lives of millions of people, this paper is a consideration, from a policymaking perspective, of practical attempts to reconcile aspects of what on the surface might appear to be irreconcilable worldviews [1].

## 2. Health in Native American and South African Societies

Healthcare indicators for Native Americans and black South Africans are significantly poorer than those of their white counterparts. For example, the difference in life expectancy between black and white South Africans is approximately 20 years [2]. Likewise, in the U.S., Native American life expectancy is lower than that of the national average, and Native American communities also demonstrate “lower health status” in a number of areas. The IHS notes:

*“American Indians and Alaska Natives die at higher rates than other Americans from chronic liver disease and cirrhosis (368% higher), diabetes mellitus (177% higher), unintentional injuries (138% higher), assault/homicide (82% higher), intentional self-harm/suicide (65% higher), and chronic lower respiratory diseases (59% higher).”* 

Given the higher health status enjoyed by most Americans, the lingering health disparities of American Indians and Alaska Natives are troubling [3]. In both regions, poverty, inadequate nutrition, high levels of violent crime, alcohol and substance abuse, and poor access to healthcare are all contributing factors, many of which can be seen to stem from colonial legacies. 

Both groups have also been affected disproportionately by HIV/AIDS. In the case of South Africa, with a prevalence rate of 12.2 percent amongst the general population (up from 10.6 percent in 2008), prevalence amongst black South Africans is 15 percent as opposed to that of 0.3 percent for white South Africans [4] (p. xxiii). This is despite a marked improvement in HIV/AIDS prevention efforts and treatment in recent years. The HIV/AIDS pandemic has also had a significant impact on Native American communities, although relatively small numbers make this impact less visible. Official statistics suggest that Native Americans are the third worst affected ethnic group in America, after African Americans and Hispanics. However, given the manner in which all people entered into the U.S. health system are (mis)classified ethnically, it is arguable that there may be significant under-reporting of the problem [5]. Furthermore, while prevalence rates are declining amongst other U.S. ethnic groups, they are continuing to rise amongst Native Americans. Evidence also shows that Native Americans infected with HIV have shorter life expectancies than those in other ethnic groups [6].

## 3. Method: Establishing a Framework for South African Traditional Healers’ Experiences 

In the U.S., there is a rich literature, much of it published by the presses of the universities of Nebraska and Oklahoma, that documents the lives of Native American “medicine men” and “medicine women”, their belief systems and their approaches to healing. These, together with observer and other anthropological accounts offer a useful—and established—body of material (see for example [7,8,9,10,11,12,13,14]) with which to contrast South African experiences. The IHS, too, is a repository for documentation detailing debates over policymaking and accommodation with respect to traditional forms of healing employed within formal healthcare structures (http://www.ihs.gov/). 

Using the Native American literature and related IHS policy as an analytic framework and site of comparison, the argument put forward here draws on original fieldwork interviews conducted with South African (and other African) traditional healers and their clients, and addresses related policies enacted by the South African government. The traditional healers interviewed for this research can be categorized broadly as *sangomas* (diviner-healers), as opposed to *inyangas* (herbalists), or faith healers linked to the traditional African churches. The perspectives put forward by the healers consulted contribute significantly to the discussion below; their willingness to share their time and experience is gratefully acknowledged. The material was gathered during the course of a series of interviews conducted in 2010 that built on previous fieldwork conducted in 2009. The interviews were conducted in four locales across South Africa, incorporating both rural and urban environments and male and female respondents of varying age, social status and education level. The identities of all informants have been anonymised. The interviews took place in Johannesburg (Gauteng Province), East London and surrounds (Eastern Cape Province), Grahamstown (Eastern Cape Province), and Knysna and surrounds (Western Cape Province). These locations were selected in the interests of interrogating the pervasiveness of engagement with traditional healing in a range of environments. Urban centres like Johannesburg and East London are contrasted with more “traditional” rural areas, such as those outside east London, which incorporate parts of the old apartheid-era “homelands” of the Transkei and Ciskei. Grahamstown and Knysna represent, for the most part, similarly rural heartlands, still influenced heavily by traditional values. In order to achieve as broad a perspective as possible, interviews were conducted with both healers and their patients and took the form of semi-structured informal interviews across individual and group sessions. While all of the traditional healers were of course working in South Africa, the nationalities of those consulted included Malawian, Tanzanian, Ugandan and Zimbabwean as well as South African. The traditional healer sample included both male and female practitioners of different levels of age, experience and standing in the community (including apprentice healers and their mentors). The patients/clients interviewed encompassed a cross-section of individuals from local communities engaged in forms of employment that included social work, management, education, domestic service, construction and tourism. The paper also draws on secondary anthropological studies focusing on traditional forms of South African healing [15,16,17,18,19,20]. 

## 4. Locating Contemporary Traditional Healing

South African and Native American traditional forms of healing are both deeply embedded and have a broad resonance that in many respects transcends the ethnic and cultural boundaries of each region. Despite the best efforts of paternalists who favoured “progress” as a way to eradicate Native American (see, for example, [21]) and South African [22] traditional healthcare perspectives, aspects of both systems have proved remarkably durable. While both of the regions concerned incorporate a number of cultural/ethnic groups that are hardly homogenous, there are sufficient commonalities across each to make at least a degree of generalisation useful. For example, while there are an estimated 4.1 million Native Americans living in the U.S., comprising 566 government-recognised tribes [3], healers from the different groups borrow extensively from each other (although some traditional healers, like the high profile ‘medicine man’ and civil rights activist John Lame Deer, have expressed concern about the amalgamation of Native American beliefs; Lame Deer has argued that, for himself at least, it was important to concentrate on his specifically Sioux traditions [9]). Similarly, in South Africa, despite there being 11 official languages and a host of often-overlapping identities, traditional healers from across the country—and as far afield as Nigeria and Uganda—are accepted as “legitimate” by local communities [1]. 

There is a tendency for traditional healing to be embraced as static, a body of knowledge/practices sealed in time. In fact, it is *“fluid, diverse and idiosyncratic”* [23] (p. xx). Claims that Native American healing has been practiced for 12,000 years (or longer, depending on when the Americas were first inhabited), intentionally or not cement this perspective [24]. It is accepted that the cataclysm wrought by infectious diseases introduced to the Americas after 1492 impacted dramatically upon the social and cultural fabric of Native American societies. Depopulation estimates range between 78 and 97 percent of the pre-Columbian population of North America [24] (pp. 324–325). By the late nineteenth century only approximately 500,000 Native Americans remained out of a pre-1492 population that may have been as high as 18 million [24] (p. 325). By extension, this undermines severely any argument that twenty-first century Native American traditions represent 12,000 years of continuity. The origins of pre-colonial South African forms of healing are similarly clouded. However, while generally conservative and rooted in the past, the traditional healing practiced today in both regions has not gone unchanged over the centuries. All systems evolve and traditional healing is no different in this respect. Colonialism engendered additional complications; knowledge and traditions have eroded in the face of centuries of instability and conflict to the point that it is difficult to say with any certainty that what is practised currently would have been recognisable to pre-colonial societies. 

Murray Last, describing traditional healing in West Africa in his seminal 1981 article, “The importance of knowing about not knowing”, argued that what can be described as a “traditional system” is, in fact, so un-systematised as to *“scarcely constitute a system, though it flourishes nonetheless. The lack of a system is seen in the disunity of traditional doctors, in their lack of a single consistent theory and in the wide variation in meaning in the medical terminology in daily use”* [25] (p. 387). In a number of respects, Last’s observations can be seen to hold when applied to other systems of traditional healing. Contemporary Native American practitioners like Lewis Mehl-Madrona—a qualified biomedical doctor as well as a traditional healer—acknowledge the fact that much of what is practiced by current healers is drawn from different sources and often based largely on “intuition” [11]. For Mehl-Madrona, *“modern shamans…pick and choose bits of different traditions, using a combination of things that work for them and their clients”* [11] (p. 135). 

The twentieth-century evolution of the peyote religion into what is now the Native American Church is an illustration of the potential for shifts and changes within traditional systems: peyotism’s move away from its central American origins—combining Catholic and Mexican ritual to form something of a pan-Native American religion—represents a quite revolutionary transformation within Native American tradition [26]. In terms of basic observances for example, peyote ceremonies usually take place in tipis even though such structures were not traditionally employed by groups of significant size like the Navajo [14]. The sweat lodge too, long viewed as a universal symbol of Native American healing (and one embraced by New Age healers), and now also incorporated as a ceremony within the Native American church, was not historically utilised by all groups. Traditional South African forms of healing have similarly seen changes, particularly in urban areas. The list of industrial ingredients found in many traditional remedies would, of course, have been wholly unknown in pre-colonial societies [27,28]. Engagement with healers from other parts of Africa also represents an evolution of traditional perspectives [29].

Roots aside, contemporary traditional healing forms an important aspect of Native American and South African perspectives on healthcare. There is a much-employed World Health Organisation statistic that suggests that 80 percent of people living in Sub-Saharan Africa make use of traditional forms of healing/medicine (see for example [30,31]). This figure was also employed with specific respect to South Africa in the *South African Medical Journal* in 2012 [32]. In 2011, President Jacob Zuma of South Africa put the figure at 70 percent [33]. While this particular statistic is something of a “factoid”, the controversy surrounding it should not be allowed to undermine the importance placed on traditional healing in Sub-Saharan Africa. In South Africa, the more considered evidence suggests a far lower but nonetheless significant proportion of adherents [34]. Similarly, with respect to Native Americans, figures for those making use of traditional healing range between 38 and 70 percent [35] (p. 670). 

## 5. Science and the Supernatural: Distinguishing between Healthcare Systems

“Traditional” and “biomedical” tend to be the terms employed in the literature to distinguish between these two systems of healthcare but this dichotomy is not unproblematic. While much of the discourse in medical anthropology and medical sociology prior to the 1980s saw a clear separation between what were perceived to be very different approaches to sickness, Bradley Stoner has shown that, globally, the division is less precisely delineated [36,37]. The reality is that practitioners and patients on both sides “dip into” the alternatives available to them; meaningful engagement can take place when the need arises. As Stoner has also demonstrated, what people want, with respect to healthcare, are options, irrespective of how these may be defined. 

Evidence from both regions suggests that the vast majority of users engage in some form of medical pluralism. Most South African and Native American traditional healers are comfortable in both distinguishing between traditional and non-traditional sicknesses, and accepting that aspects at least of the latter are best treated through recourse to biomedicine. It is also clear that Native American traditional healers themselves see nothing problematic with engaging personally with biomedicine when necessary—Conley [10], Mohatt [13] and Langley [14] all record conversations with healers freshly discharged from their local hospitals. 

Until recently, analysts saw the lines dividing the two systems as being fairly clear cut (and at odds with each other), especially on matters involving the supernatural. Bluntly, the European and North American perspective was that biomedicine was based on science while traditional healing was not—traditional healers could not be viewed as “doctors”. Anthropologists like Erwin Ackerknecht, writing in the 1940s, viewed Native American healers as having been *“the antagonist(s) of the physician for centuries”* [38] (p. 22). As Ackerknecht saw it, healers were *“ancestor[s] of the priest”* rather than of the modern biomedical practitioner. In colonial Africa, “witchdoctors” were dismissed as being representative of the “dark continent” that colonialism sought to displace [39]. 

At the same time, appeals to the healing powers of supernatural forces are not as divorced from a contemporary biomedical perspective as many operating within a “rationalist” mindset would like to admit. Most biomedical hospitals have chaplains of various faiths attached and prayer is employed regularly by users of biomedical institutions as a complement to treatment (a survey suggests that up to 70 percent of Americans believe that prayer can help to cure sickness) [40] (p. 577). Moreover, although attempting to quantify the impact of prayer on those who are sick is methodologically controversial, evidence from a small number of randomised tests suggests that it may be possible to identify a positive effect. A study focusing on coronary patients in the U.S. *“found that supplementary, remote, blinded, intercessory prayer produced a measurable improvement in the medical outcomes of critically ill patients…further studies using validated and standardized outcome measures and variations in prayer strategy are warranted to explore the potential role of prayer as an adjunct to standard medical care”* [41] (p. 2278).

A study amongst HIV/AIDS patients in the U.S. during the 1990s suggested that, although CD4 counts remained unaffected, prayer resulted in better indicators for disease progression, decreased medical utilization, and improved psychological well-being [42] (p. 357). The authors argued that *“science does not require a known mechanism to prove the existence of a phenomenon...for years no one knew how colchicine, morphine, aspirin, or quinine worked, yet they were known to be effective”* [42] (p. 362).

Such evidence is, however, far from conclusive. Scientists and biomedical practitioners continue to debate hotly the extent to which the impact of prayer can be studied objectively. Be that as it may, it can be taken that many patients and practitioners within the biomedical system believe in the power of supernatural intercession [43]. Critically, as an extension, the majority of patients also see no conflict in requesting supernatural intercession whilst being treated within a healthcare framework that offers no ontological space for the supernatural. 

## 6. Biomedical and Traditional Perspectives: Establishing Common Ground?

Native American and South African forms of healing should not be understood as nascent forms of biomedicine that, given time and space, will evolve into something akin to the western model. As Dickinson outlines, despite ongoing focus in this regard, the prospect of a *“bilingual medical-traditional dictionary”*, capable of translating traditional perspectives into biomedical parlance represents something of an impossibility [23] (p. xx). Rather, these examples of traditional healing form alternative systems of healthcare. In both instances, unlike in the pathology-focussed biomedical model, traditional forms of healing rely heavily on aspects of the supernatural with respect to both diagnosis and treatment [29,44]. Here, the terms “healer” and “medicine” extend more broadly than they do within the general biomedical paradigm. A group of male Lakota healers, when questioned as to how they should be formally identified, after rejecting the terms *“medicine man”*, *“holy man”* and *“interpreter (for the spirits)”*, finally settled on a term that translates as *“the man that fixes”* [13] (pp. 13–14). 

Alongside questions regarding training and expertise, it is the supernatural aspect that so many in the biomedical sector find difficult to accommodate within a “rational” scientific framework. For example, traditional healers from both traditions are generally called to the profession at a young age through dreams and visions [10,11,29,45]. Every healer interviewed for this study told of a similar “calling”: from childhood, vivid and disquieting dreams in which their ancestors tried to establish lines of communication with them [29]. Many, although not all [29], who come to the profession are either illiterate or have had little formal schooling [9,46], and much of their “power” is derived from the strength of their relationships with the “spirit world” and/or their ancestors. Consequently, while forms of what might equate to biomedicine are practiced by traditional herbalists [38] who utilise plants to treat ailments, most medicines and treatments are “revealed” through supernatural engagement [11,13,29]. 

Illness in both Native American and South African traditions is generally understood to be less the result of pathogens or physiological changes and rather the result of supernatural interventions brought about through either one’s own spiritual missteps or malevolent intent on the part of others. One’s own provoking of supernatural displeasure can come about through the violation, intentional or otherwise, of taboos, obligations and responsibilities. Malevolent intent on the part of others, on the other hand, involves witchcraft: the deliberate calling forth of negative supernatural intervention in another person’s life [15,38]. In both traditions it is witchcraft that is understood to play an often central role in the causation of illness. As a result, illness is rarely viewed as transmittable; witchcraft is nearly always person- (or, occasionally, family-) specific [10,14,29]. The invoking of witchcraft, as perceived across both cultures, generally originates out of jealousy and a desire to see a successful person brought low. Witchcraft is understood to bring about general misfortune, financial problems, alcohol and/or substance abuse, relationship and personal issues, and, critically, ill health. As a number of informants contributing to this study detailed, bad luck is, essentially, no matter of chance; both good and bad fortune are shaped by the supernatural [29]. Resolution of bad fortune, therefore, is also understood to require supernatural intervention, usually invoked through ceremonies and rituals. Medication, if required, is determined on the basis of supernatural direction rather than pharmaceutical benefit—for example, the smoking of blessed cigarettes is a common prescription within Native American healing [11,47]. 

The emphasis on the supernatural has, from the initial stages of European intervention, resulted in accusations of “quack medicine” and of “charlatans” preying on the weak and gullible. More recently, that clinical trials have found few traditional medicines to impact positively on pathogens has served to corroborate this earlier perspective. South Africa is just one of the African countries where this has led, in part, to governmental attempts to regulate and control the traditional sector, through the provision of certification for “genuine” healers (Ghana, Nigeria, Burkina Faso, Democratic Republic of Congo, Guinea, Madagascar and Mali have legislation governing the registration of traditional medicines [48]). However, attempts by both policymakers and the biomedical community to codify what is and what is not traditional healing reveals a fundamental misunderstanding of what it is and how it is practiced. For example, the idea of charlatan healers sits uncomfortably within both cultures—in general, failure to find a cure is often viewed as an indication not of fraudulence or misdiagnosis on the part of the traditional healer, but rather of the strength of the supernatural forces aligned against the patient. In such cases, it thus becomes a matter of seeking out a more powerful healer. In South Africa, it tends to be understood that “genuine” healers will only request payment once the patient is healed. A healer demanding payment upfront may sometimes be deemed suspect [29]. 

The gulf between biomedical and traditional cosmologies is undeniably wide, as are the respective approaches to diagnosis, patient care, and treatment; double-blind testing and laboratory-based demonstrations of efficacy are inadequate tools for validating diagnoses acquired through communication with the spirit world. Medical anthropologists have, in the past, sought to address these differences by distinguishing between the conceptualisation of “disease” and “illness”, and “curing” and “healing”. Curing is, in essence, a largely biological process that results in the removal of disease from the body. Illness is viewed generally as a more psychosocial condition involving spiritual or mental health aspects in need of healing (for a more detailed discussion see [49]). While the utility of this dichotomy is much debated, it tends to be the case that biomedicine is focused on the curing of disease while traditional healing is inclined to be more concerned with illness. In basic terms, the two systems are not necessarily attempting to achieve the same goals or outcomes, which can be confusing to outsiders, particularly given that the language used to describe both processes is often seemingly interchangeable. In part this is due to the fact that the lexicons of traditional cosmologies often translate poorly into English (and other European languages), resulting in the obscuring of major epistemological divides. When traditional approaches are described in English terms, with the biomedical associations of the latter, it can create illusions—if not aspirations—of equivalence: in English, many traditional healers themselves reach for what is largely biomedical terminology in order to describe their work to outsiders. Traditional healers marketing themselves in urban South Africa often adopt the titles “doctor” or “professor”. They routinely receive their patients in offices, with waiting rooms staffed by receptionists, in much the same way as biomedical general practitioners would. However, as Waldram points out, traditional healers’ interpretations of biomedical terminology are rarely considered [49]. 

## 7. The Advantages of Traditional Healing for Patients 

Mehl-Madrona describes how, during his biomedical training, management demanded that he see at least three patients per hour [11]. This is by no means unusual within biomedicine; doctor-patient interaction is generally kept to a minimum. The UK National Health Service (NHS) suggests that a GP will see, on average, between thirty and forty patients per day [50]. In Native American and South African communities, where biomedical healthcare personnel and infrastructure can both be limited, practitioner-patient “face time” can be somewhat perfunctory. The resultant feelings of alienation and frustration experienced by patients under these circumstances can be exacerbated when practitioners are unable to engage patients in their own languages. Traditional healers, on the other hand, tend to spend a great deal of time with their patients, with some Native American ceremonies extending across a number of days [10,12,14]. Some intensive therapies have been known to involve daily contact between healer and patient for over a month [13]. Amongst the Navajo, complex ceremonies can involve up to a hundred hours of ritual chanting on the part of the healer, and can also require the participation of the patient’s entire extended family [51]. In fact, what stands out when comparing the two systems, is that traditional healing, unlike that of biomedicine, is often communal rather than private. In all cases, patients are asked to be part of the process in the interests of contributing to their own healing. Furthermore, due to the ways in which the supernatural is perceived to impact on people’s health, treatments and ceremonies are tailored to the requirements of individuals, as is any prescribed medication [11]. Likewise, traditional forms of healing in South Africa are unhurried and deeply personalised, with treatment being specifically tailored to each individual [29]. South African traditional healers see far fewer patients than their biomedical counterparts, offering in-depth treatment that includes counselling for both individual patients and their families [46]. South African traditional healers, like their Native American counterparts, also frequently travel significant distances to treat patients in their own homes [52]. Traditional healing is therefore a highly individualised and interactive experience for patients, in which healers are facilitators. This differs substantially from the expert-driven approach central to biomedicine.

Engagement with the supernatural can also enable people to find a measure of meaning in their suffering, even if these same individuals are prepared to accept the “germ theory” of disease as the immediate cause of their illness [53]. As with biomedical practitioners, traditional healers identify what is wrong with their patients—the naming of the illness presented is viewed as a significant aspect of the treatment process. In this respect, the divergence between the systems occurs on the basis of the significance surrounding a patient’s experience of ill health that is offered by traditional healing. In contrast, biomedicine has little to offer patients in terms of explanations for their misfortune—diseases are contracted randomly (at least in part) against the background of a largely disinterested universe. In this respect, a “witchcraft paradigm” [16,17] offers a degree of solace to patients; their suffering can be seen to have been caused by malevolent forces deployed against them, usually out of jealousy, rather than having come about through random infections and mutations. Jealousy-as-causation was a recurring theme within the majority of the discussions on witchcraft that arose during this study, with both patients and healers in broad agreement on this aspect [29]. At the same time, within such a worldview, it must be emphasised that for every victim of witchcraft there must be a perpetrator [10,14,29]. Instances of retaliation—often violent—against “witches” by communities in South Africa are well documented [54]. In 1995, as a result of nearly 150 deaths the preceding year in Northern Province, South Africa, a commission of inquiry was established to investigate witchcraft-related murders [55]. In 2007, in response to similar levels of violence, Mpumalanga Province, South Africa, passed the Witchcraft Suppression Bill that made the identifying or “sniffing out” of witches a criminal offence. While there is less documented detail regarding the negative social effects of a witchcraft paradigm on Native American communities, retribution—usually supernatural—is often implied [10,14]. 

## 8. Bringing the Traditional and Biomedical Sectors Closer Together

Studies in the U.S. have shown that traditional healing techniques have proved most effective in treating alcohol and drug addiction, often associated as these addictions are with physical, sexual and domestic violence, and HIV/AIDS-risk behaviours. The traditional healing approach has also been proved to be effective in the treatment of mental health disorders such as depression. In the 1950s, the IHS was already beginning to employ “culturally appropriate” forms of engagement with patients, bringing traditional healers into the formal system in the interests of promoting better “therapeutic outcomes” [56]. In this regard, the first director of the IHS, appointed in 1953, James “Ray” Shaw, saw improving ties with traditional healers as an important aspect of the agency’s mission [57]. Once established, the relationship between the IHS and traditional healers became (to a degree) reflexive. For example, the head of mental health services for the IHS in the 1970s, Robert Bergman, describes the establishment, by Navajo healers, of a “school for medicine men” in which he taught classes on biomedical practice and psychiatry and was, in turn, moved to refer his own patients to local traditional healers [51]. Fostering cooperation has remained an important part of the IHS mission, especially in the wake of the 1976 Indian Health Care Improvement Act, which offered a far greater degree of self-determination to Native American communities with respect to the running of the IHS, and the 1978 American Indian Religious Freedom Act, which afforded protection for Native American religious beliefs. Reflecting on the impact of the Act in 1994, an IHS policy paper stated that:

*“The Indian Health Service (IHS) recognizes the value of traditional beliefs, ceremonies, and practices in the healing of body, mind, and spirit. The IHS encourages a climate of respect and acceptance in which traditional beliefs are honored as a healing and harmonizing force within individual lives, a vital support for purposeful living, and an integral component of the healing process. It is the policy of the IHS to facilitate access to traditional medicine practices, thereby protecting the right of American Indian and Alaska Native people to their beliefs and health practices as defined by the tribe’s or village’s traditional culture”*.

[58]

At the same time, it would appear that dissatisfaction with biomedical treatment is not the driving force behind engagement in medical pluralism and neither is the cost [29,59]. Studies, from as far back as the 1952 Many Farms project targeting tuberculosis amongst Navajo communities, suggest that patients could be persuaded to undergo simultaneous biomedical and traditional treatments, on the basis of what was accepted as the complementary potential of a combined approach [56,60]. Contemporary Native American healers themselves relate how they use their powers to assist biomedical practitioners in their work, of which an example might be invoking their spirit guides to work with surgeons in operating theatres [13]. Furthermore, evidence suggests that, with respect to biomedical regimes, self-compliance remains unaffected by consultation with traditional healers [59].

Notable too are the costs involved in both Native American and South African traditional healing, especially when compared with those of biomedical alternatives. A 1998 study amongst Navajo communities found the average cost of a traditional treatment to be approximately $400, a significant figure given the high rates of poverty and unemployment within these communities; IHS services are provided free of charge. Some traditional treatments were reported to cost as much as $3000 [59]. The South African situation is similar. South African biomedical care, although often limited in rural areas, is free; intensive traditional treatments, often requiring animal sacrifices, can currently cost as much as R5000 ($470) [29], when the average unskilled wage is just R10 ($1) per hour.

Despite a history of chronic underfunding—the IHS has traditionally been funded to a much lower degree than other Federal programmes like Medicare and Medicaid [61]—the IHS’ remit means that it can offer a more tailored response to its patients than that which is available in South Africa. Like the IHS, the South African healthcare system is underfunded and understaffed. Critically, the South African system is also less flexible in its approach. As a national healthcare provider serving a population of 54 million [62], it is in many respects unsurprising that IHS-style engagement between the biomedical and traditional healthcare sectors has had little space in which to evolve. In South Africa’s case, while gradual change is now beginning to take place, the relationship between biomedical and traditional practitioners remains one governed largely by misunderstanding. That this climate exists is due at least in part to the fact that no institution along the lines of the IHS is present in South Africa. In the IHS, as an institution geared specifically to the treatment of a far smaller group of people drawn from a relatively common cultural background, the potential for accommodation between the sectors has been far greater. 

Funding and diversity issues notwithstanding, differences in the biomedical/traditional climates of the U.S. and South Africa are also the result of policymakers framing official narratives on traditional healing in different ways. With respect to the Native American experience, policymakers’ efforts to codify the different spheres “owned” by the two healthcare systems have resulted in these being accepted as different but complementary (although this is not to suggest that some mutual suspicion does not exist). In South Africa, the two systems are usually presented as being in competition with one another. That this (often false) dichotomy has remained entrenched is due largely to the focus placed on traditional healers and traditional medicine by the Mbeki government (1999–2008), in reaction to its failure to deal with the spiralling HIV/AIDS pandemic of the period. At this time, a shortage of biomedical practitioners also meant that the government was under pressure on healthcare issues more generally (For a middle-income country such as South Africa, the World Health Organisation suggests a doctor-to patient-ratio of 180:100,000. The South African reality is closer to 50:100,000, with rural areas being particularly poorly served [46]. Traditional healers outnumber biomedical practitioners by almost 10:1 [1]). Affording equivalence to the traditional sector was seized on by policymakers as a way of killing two birds with one stone. 

As is well documented, President Thabo Mbeki was “sceptical” about the links between HIV and AIDS, being persuaded by much of the denialist “science” on the subject. The result, driven by his determination to present his position as being one based on resistance to imperialist values, was an increased focus on traditional medicine as a potential “African solution to an African problem”. This message had traction because, as Dickinson points out, while South Africa is not a traditional society, *“it still retains much traditional belief”* [23] (p. 26). Furthermore, during the early days of the pandemic, when mass South African access to the new biomedical response that was ART was a remote possibility, those who were HIV-positive sought out alternative treatment regimes, many of which included forms of traditional healing. Corinne Squire’s “narrative approach” to understanding the pandemic in South Africa highlights how, during the Mbeki period, people living with HIV/AIDS (PLWHA) set about supporting themselves through the construction of their own AIDS narratives [63]. 

The government, for its part, sought to legitimise its preferred counter-narrative through the formalisation and regulation of the traditional healthcare sector, framing it as part of Mbeki’s “African Renaissance” [64]. The subsequent 2008 Traditional Health Practitioners Act ignited significant discussion on, amongst other issues, the rights of traditional healers to write legally-valid “sick notes” for workers, and whether patients could claim for traditional healing expanses on their medical insurance [32]. Albeit for different reasons, Mbeki’s successor, Jacob Zuma, whose popularity rests at least in part on his staunch support of traditional values, has maintained emphasis on the continued mainstreaming of traditional healing: *“(o)ur commitment as government is to bring* *traditional medicine into the mainstream of health care, appropriately, effectively and above all safely”* [33]. 

While there has been some considerable movement towards accommodation on the part of certain South African practitioners (on both sides of the divide), biomedical practitioners have been accused of conducting what amounts to medical missionary work rather than attempting to truly accommodate traditional cosmologies [1]. For example, while traditional healers have been strongly encouraged to press patients they suspect of being HIV-positive to be tested, and many have expressed themselves content to do so [29,65], surveys suggest that biomedical practitioners are, on the other hand, reluctant to recommend traditional healers to patients [46]. South African interaction is, in effect, the “education” of traditional healers into the biomedical perspective, rather than a “meeting of minds”. 

## 9. Traditional Healing and the Treatment of HIV/AIDS

Debates regarding value systems, cultural sensitivity and ideological accommodation become highly charged when, as is the case with South Africa, literally millions of lives continue to be threatened by HIV/AIDS. In addressing HIV/AIDS within Native American communities, the use of traditional healing has been most successfully employed in treating illnesses involving substance and alcohol abuse (which have in turn have been shown to exacerbate HIV/AIDS-associated risk behaviours). Native American traditional healing has not been presented as an alternative form of HIV/AIDS treatment, and neither has it been viewed as such in the wider community. In South Africa this has not always been the case, meaning that lines are often blurred [29]; for this reason, levels of HIV/AIDS education remain unacceptably low [23,66]. 

In contrast to illnesses that have shown themselves to be responsive to traditional healing, HIV/AIDS and its treatment offers little doubt with respect to efficacy, regardless of how the latter may be defined [49]. The indisputable success of ART has made the case for biomedical treatment irrefutable. With PLWHA increasingly able to live into old age so long as they have adequate treatment, the importance of engaging with ART cannot be overemphasised. This, however, reduces dramatically the scope for patients to engage in medical pluralism. Traditional approaches cannot treat HIV/AIDS. This not to say that that traditional healing cannot have a positive effect on the conditions and symptoms affecting PLWHA—especially with respect to efforts to assign meaning to suffering—but attempts to suggest that comparable efficacy is a matter of interpretation is problematic. The human cost in not engaging with biomedical HIV/AIDS treatment is both high and undeniable; researchers from the Harvard School of Public Health suggest that, as a result of the South African government’s failure to roll out ART between 2000 and 2005, *“more than 330,000 lives or* *approximately 2.2 million person years were lost”* [67]. The same study estimated that during this period 35,000 babies acquired HIV via mother-to-child transmission, something that might have been averted through the administration of the ARV nevirapine [67]. Of equal concern is evidence published in the journal *AIDS* in 2006 that pointed to low levels of awareness of appropriate HIV/AIDS treatments amongst South Africans [66]. It was clear from this study that many South Africans viewed ART as simply one of a number of HIV/AIDS treatment options. The authors of the study argued strongly that *“if antiretroviral agents are to compete more successfully in the therapeutic continuum, there needs to be explicit recognition of, and further strategies to counter, the attraction of alternative therapies for patients and the systematic promotion these treatments receive”* [66] (p. 1977). While the Zuma government has been far more forthcoming with respect to addressing the pandemic than its predecessor, it is clear that serious confusion remains with respect to HIV/AIDS. The fact that a 2014 report shows that, two decades after the democratic transition, South African prevalence rates continue to rise as a result of, in part, an estimated 469,000 new infections in 2012 (the year of the study) alone, suggests that the pandemic shows little sign of easing [4] (p. xxix).

The “obvious” superiority of biomedicine in this particular instance, combined with statistics such as the above, have led to a sometimes fractious debate over the value and place of traditional forms of healing in South Africa. As stated, Native American prevalence rates have tended to be driven by risk behaviours associated with alcohol and substance abuse, and prevention programmes have been developed with the intention of addressing these behaviours [68]. Groups like the Navajo AIDS Network (NAN), a non-profit organisation that works with the IHS, have established structures that allow for culturally-appropriate systems of treatment for PLWHA, including traditional healing. One of the services offered by the NAN helps PLWHA identify and fund treatment with traditional healers. At the same time, a notable outcome of the programme has been increased adherence to biomedical regimes [69]. What is clear, in this instance, is that traditional healing is not being proffered as an alternative to ART. Rather, it is seen as a vehicle for both the provision of culturally-sensitive forms of HIV/AIDS education and the encouragement of prevention behaviours. Where South Africa is concerned, traditional remedies are frequently prescribed by healers as ART alternatives. One of the more publicised of such cases was that of Zeblon Gwala and his *uBhejane* (rhino) herbal “cure”, which developed a devoted following in KwaZulu-Natal in 2006, the South African province with the highest levels of HIV/AIDS in the country. Gwala, a truck driver turned traditional healer, claimed that the treatment was revealed to him in a dream by his late grandfather who had, in turn, also been a traditional healer [70]. In promoting his remedy, he claimed that it *“increases your CD4 count and reduces the viral load until it disappears”* [71]. Critically, Gwala also advised all patients taking his treatment to discontinue ART. An additional concern to AIDS campaign groups was that Gwala received active support from, amongst others, Mbeki’s Minister of Health, Mantombazana Tshabalala-Msimang and other political elites. When subjected to clinical trials in 2005, the Medical University of South Africa found *uBhejane* to have no positive effect on the treatment of HIV/AIDS [72]. AIDS activists argued strongly that official support for *uBhejane* (along with other traditional treatments), created confusion in the minds of South Africans as to the value of ART [46,73].

While there have been attempts by the national and provincial governments in South Africa to train traditional healers in HIV/AIDS awareness, the funding allocated to such efforts has been limited and, as a result, the rollout of appropriately trained healers has been inadequate [74]. Much of the training that has occurred has been left to NGOs. This is problematic; evidence suggests that many traditional healers remain poorly informed about HIV/AIDS. A study published in the *South African Medical Journal* in 2004 estimated that only 6.25 percent of traditional healers had undergone government-funded HIV/AIDS awareness training [74]. Furthermore, where government training has been provided, the results have, at times, been disappointing, with significant numbers of healers still professing a belief that HIV/AIDS can be cured [46]. Additional studies have also pointed to the continuation of HIV/AIDS risk-associated practices, such as the use of unsterilized equipment for the administering of enemas and the use of single blades for the scarification of multiple patients [75]. That said, the *South African Medical Journal* study showed that traditional healers were increasingly demonstrating a willingness to promote condom usage and to stress the dangers of unprotected sex with multiple partners [74].

## 10. Conclusions

This paper is not intended as a study of either the efficacy of traditional healing or alternative belief systems. Rather, it is an attempt to move beyond the usual calls for better understanding between traditional healers and biomedical practitioners in South Africa. These generic calls do little to shift the debate past the above very basic point. Using the Native American experience as an analytical framework, the paper has sought to understand how meaningful accommodation might be accomplished in an instance where a degree of this has already been achieved. The evidence suggests that the IHS, in acknowledging the importance of culturally-sensitive approaches to HIV/AIDS treatment, has found room for traditional healers within the formal structures of the system. In stressing the complementary potential of traditional and biomedical systems, IHS policymakers have eased the historical tension between the two sectors. In the South African case, the discourse reflects many of the unresolved tensions that continue to emanate from the colonial and apartheid legacies; an adversarial tone is immediately obvious in many aspects of the debate. The HIV/AIDS pandemic in particular, and the politicised narrative that emerged from it, has served to entrench yet further the distance between the two systems. Consequently, in South Africa, the two systems are often presented as an “either-or” scenario. If the South African government is serious about its engagement with the merits of traditional healing, as opposed to using the sector to offset deficiencies in public health spending or to redress aspects of the injustices of apartheid, then the current dichotomy of medical choice is an issue that must be addressed. In this regard, the IHS model offers a useful starting point. Importantly, for all concerned in the policymaking sphere, there needs to be a shift away from any attempts to create equivalence, towards an acceptance of each system on its own merits. Dickinson’s *“bilingual medical-traditional dictionary”* analogy is useful in this respect [23]; it is only the abandoning of the contemporary emphasis on syncretism that will enable further progression towards a viable form of accommodation. The identification of biomedical equivalence, double-blind clinical trials, and attempts at the codification of traditional healing (and traditional medicine) and its best practice, misses the point. For its proponents, the value of traditional healing lies elsewhere. In essence, the evaluation of traditional healing within an assessment framework that is itself a product of the evolution of the biomedical sector condemns the traditional sector from the outset; its practice will always be viewed by stakeholders within the formal healthcare sector as, at best, misinformed. Open acknowledgement of the integral aspects of much traditional healing that are, at present, downplayed in the interests of shoring up its position within a biomedical framework—that it is not “rational”, that it is not based on scientific principles, and that it is not falsifiable—represent, somewhat counter-intuitively, the best means of bringing the sectors closer together.
